# Surveillance of healthcare-onset clinical cultures using whole-genome sequencing reveals hidden nosocomial transmission

**DOI:** 10.1017/ash.2023.340

**Published:** 2023-09-29

**Authors:** Mohamad Sater, Emma Briars, Connor Parrish, Ian Herriott, Kathleen Quan, Keith Madey, Julie Shimabukuro, Linda Dickey, Shruti Gohil, Alfred Wong, Talia Hollowell, Allison Brookhart, Alison Gassett, Miriam Huntley, Susan Huang, Cassiana E. Bittencourt

## Abstract

**Background:** Traditional hospital outbreak-detection methods are typically limited to select multidrug-resistant pathogens in a single unit, which can miss transmission of many medically important healthcare-transmissible pathogens. Whole-genome sequencing (WGS) enables comprehensive genomic resolution for accurate identification of clonal transmission. Previously, lack of scalability limited the use of WGS for hospital surveillance. **Methods:** We conducted prospective surveillance of select bacteria from all inpatient clinical cultures plus all bacteria from clinical cultures from ICUs and oncology units at the University of California Irvine (UCI) Clinical Microbiology Laboratory from September 2021 to February 2022. Due to pandemic stressors, this pilot test was a prelude to a real-time demonstration project. Its goal was to demonstrate the efficiency and scalability of the WGS platform when receiving samples monthly and analyzing results quarterly without the intent for real-time response. Bacterial isolates slated for discard were collected weekly and sent monthly to Day Zero Diagnostics for sequencing. In total, 1,036 samples from 926 patients were analyzed for genomic relatedness, a scalable and automated analysis pipeline already in use for rapid (days) characterization of genomic-relatedness in small and large sets of isolates. Mapping and SNP calling was performed against high-quality, best-match reference genomes. Sets of samples with pairwise distance of 2 persons with genomically related isolates and were denoted as “clusters.” Separately, we also investigated within-patient diversity by quantifying the genomic relatedness of isolates collected from individual patients. **Results:** Isolates represented 28 distinct species. We identified 10 *Escherichia coli* clusters (range, 2–4 patients; median, 2 patients), 2 *Klebsiella pneumoniae* clusters (range, 2–4 patients), and 1 *Enterococcus faecium* cluster (3 patients). All but 1 involved genomically matched isolates from multiple hospital locations. There were 4 *Escherichia coli* ST131 clusters spanning 4 months, including 1 with 4 patients across 3 different hospital locations. At a species level, there were distinct differences between the observed SNP distances between samples isolated from the same versus different patients (Fig. 1). All identified clusters had not been flagged by routine outbreak detection methods used by the UCI infection prevention program. **Conclusions:** Comprehensive WGS-based surveillance of hospital clinical isolates identified multiple potential transmission events between patients not in the same unit at the time cultures were taken. Combining WGS detection and real-time epidemiologic investigation may identify new avenues of transmission risk and could provide early warnings of clonal transmission to prevent larger outbreaks. High-volume surveillance of hospital isolates can also provide species- and context-specific clonality.

**Figure:**
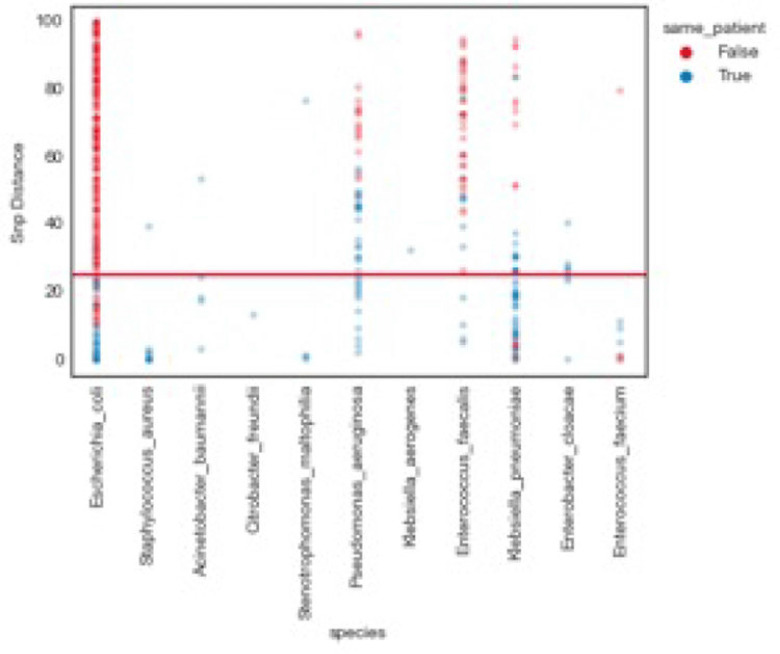

**Financial support:** This study was funded by Day Zero Diagnostics.

**Disclosures:** None

